# Distribution of Micro-Nano PS, DEHP, and/or MEHP in Mice and Nerve Cell Models In Vitro after Exposure to Micro-Nano PS and DEHP

**DOI:** 10.3390/toxics11050441

**Published:** 2023-05-07

**Authors:** Jie Han, Jun Yan, Kang Li, Bencheng Lin, Wenqing Lai, Liping Bian, Rui Jia, Xiaohua Liu, Zhuge Xi

**Affiliations:** Tianjin Institute of Environmental and Operational Medicine, Tianjin 300050, China

**Keywords:** micro-/nanoplastics PS, combined exposure, DEHP, tissue distribution, cell uptake

## Abstract

Polystyrene (PS) and di-(2-ethylhexyl) phthalate (DEHP) exist widely in the environment. However, their distribution in organisms remains unclear. We used three sizes (50 nm, 500 nm, and 5 μm) of PS and DEHP to study the distribution and accumulation of PS, DEHP, and mono(2-ethylhexyl) phthalate (MEHP) in mice and nerve cell models (HT22 and BV2 cells) and their potential toxicity. Results showed that PS entered the blood of mice, and the distribution of different particle sizes in different tissues was different. After the combined exposure to PS and DEHP, PS carried DEHP, which significantly increased the DEHP content and MEHP content and the highest content of MEHP was in the brain. With the decrease in PS particle size, the contents of PS, DEHP, and MEHP in the body increased. The levels of inflammatory factors were increased in the serum of the PS or/and DEHP group. In addition, 50 nm polystyrene can carry MEHP into nerve cells. These results suggest for the first time that PS and DEHP combined exposure can induce systemic inflammation, and the brain is an important target organ of PS and DEHP combined exposure. This study may serve as a reference for further evaluation of the neurotoxicity induced by combined exposure to PS and DEHP.

## 1. Introduction

Plastic products have sharply increased in production owing to their low price, convenient use, and wide applications in our daily lives. In the past 70 years, global plastic production has significantly increased from 1.5 million tons in 1950 to approximately 367 million tons in 2020 [1]. Plastics can be degraded into small fragments, such as microplastics and nanoplastics, through physical and chemical processes [2]. Micro-nano plastics (MNPs) are ubiquitous in natural ecosystems, such as terrestrial, aquatic, and atmospheric systems, due to the high consumption and improper disposal of plastic products. Compared with the increased output of plastic, most countries recycle only a small part of plastic waste [3]. Ingestion is one of the main routes of human exposure to MNPs. Specifically, MNPs can enter the human body through the food chain and threaten human health. Microplastic particles have been detected in seafood (e.g., fish [4,5], shellfish [6], and crustaceans [7]) and agricultural products (e.g., alcohol [8], sugar, and honey [9]). Plastic products, such as tea bags [10], milk bottles [11], and bottled water [12], also release microplastics during use. In addition, microplastics have been found in human feces, the colon, and the placenta [13,14,15]. Therefore, understanding the effect of microplastics on human health is essential. Studies have found that MNPs exposure leads to local inflammation in the intestinal tract and changes in the microbiota in mice, thereby triggering metabolic disorders and affecting the fat metabolism of the liver, leading to renal tissue inflammation, testicular structure destruction, and abnormalities in sperm [16,17,18,19]. In addition, it also leads to learning and cognitive disorders in mice [20].

Given their large specific surface area and strong surface hydrophobicity, microplastics are easy to adsorb and enrich environmental organic pollutants [21,22]. In addition, some additives, such as plasticizers and antioxidants, are added during the molding of plastic products to improve the properties of polymers and increase the durability and sustainability of plastic products [23]. These plastic additives are released into the environment as microplastics during the crushing of plastic products [24]. During the co-exposure of microplastic and organic pollutants, microplastics may transfer organic pollutants to organisms and promote the accumulation and toxicity of both pollutants in organisms [25]. MNPs and environmental pollutants exert more significant biological toxicity and bring higher health risks when present together than when present alone [26,27]. For example, Guilhermino et al. found that microplastics and the antibacterial agent florfenicol accumulate in the body after being ingested by puffer fish from the water environment. Their combined neurotoxicity and oxidative damage exceed their individual effects [28]. The combined action of microplastics and iron promotes iron accumulation in the brain and aggravates iron death, thus causing serious damage to cognitive function [29].

Di-(2-ethylhexyl) phthalate (DEHP) is a typical organic pollutant in the environment; it is the most widely used plasticizer among phthalate esters and is used to make various consumer goods. DEHP in food mainly comes from packaging materials [30]. It has also been detected in vegetables grown in DEHP-plastic film greenhouses [31]. In addition, this pollutant can be detected in various biological samples, including breast milk, blood, urine, placenta, amniotic fluid, and female follicular fluid [32,33,34]. DEHP release into the environment threatens the ecosystem and human health. DEHP has been detected in water bodies worldwide, such as lakes and rivers, sediment, urban sewage systems, and even bottled water [35]. DEHP is a metabolite with a short half-life. It has a distribution half-life of 1.886 ± 0.480 h and an elimination half-life of 36.473 ± 8.324 h, indicating that DEHP is absorbed quickly but eliminated slowly in mice. At 2 h after the oral administration of DEHP, its primary metabolite, mono(2-ethylhexyl) phthalate (MEHP), had the highest concentration among all DEHP metabolites. Approximately 67% of DEHP is metabolized into urine after 24 h, and 3.8% is metabolized into urine on the second day [36]. MEHP is the primary metabolite [37,38] and the main biotoxic metabolite of DEHP [39,40]. MEHP can damage the testicular structure, inhibit follicular development, induce placental apoptosis, lead to lipid metabolism disorders, damage the structure of the liver, and produce neurotoxicity in mice [41,42,43,44,45,46].

Polystyrene (PS) has a high yield, wide distribution, and strong adsorption capacity for hydrophobic organic pollutants. In addition, PS has a stronger adsorption capacity for phthalate esters when compared with polyvinyl chloride and polyethylene [47]. Therefore, MNPs and DEHP are likely to co-exist and cause compound pollution and exposure. Inflammatory is an important mechanism of mammalian cytotoxicity. Exploring the carrier, distribution, and inflammatory response of PS-loaded DEHP in organisms is important to evaluate the biological toxicity of MNPs and understand the health risks of MNPs and environmental pollutants. However, the distribution of PS-loaded DEHP and cumulative changes in tissues, organs, and cells of organisms exposed to micro-nano PS loaded with DEHP, and the inflammatory response in vivo remain unclear. Thus, this study aimed to systematically analyze the effects of combined sub-chronic exposure to micro-nano PS-loaded DEHP on the distribution and accumulation of PS, DEHP, and MEHP in major tissues of mice, in two nerve cell models in vitro, and the inflammatory response in vivo.

## 2. Materials and Methods

### 2.1. PS and DEHP

In this study, spherical PS (ρ = 1.05 g mL^−1^) with particle sizes of 50 nm, 500 nm, and 5 μm were purchased from Zhongke Leiming (Beijing) Science and Technology Co., Ltd. PS was uniformly dispersed in pure water at a concentration of 50 mg mL^−1^. According to the manufacturer’s information, no additives were added into the PS dispersion solvent. The PS was divided into two forms: non-fluorescent and fluorescent. Nile red was used for labeling, with the excitation and emission wavelengths at 530 and 635 nm, respectively. The morphology of PS was characterized under a scanning electron microscope (SEM; JSM-7600F, Japan Electron, Tokyo, Japan). The composition of PS without any plastic plasticizer was confirmed using Fourier transform infrared spectroscopy (FTIR-1500, Tianjin Chinese Walker, Tianjin, China). The hydrodynamic diameter (d_h_) and Zeta potential of PS were measured using dynamic light scattering (DLS) and electrophoretic light scattering analyzer (ELS) (Zetasizer Nano, Malvern Instruments Ltd., Melbourne, UK). We verified whether the fluorescence-labeled PS could emit stable red fluorescence using a laser confocal microscope (TCS SP5, Leica, Nussloch, Germany). DEHP and MEHP were purchased from AccuStandard (New Haven, CT, USA). A 50 mg mL^−1^ amount of DEHP solution was prepared with methyl alcohol (purity > 99.9%) and stored at 4 °C.

### 2.2. In Vitro Adsorption of DEHP by PS

The adsorption of DEHP by PS in vitro was tested to simulate the adsorption of compounds by micro-nano PS. The mixed solution containing 5 mg mL^−1^ PS (50 nm, 500 nm, and 5 μm) and 50 μg mL^−1^ DEHP was shaken at 20 °C for 24 h and then filtered through a 20 nm polytetrafluoroethylene membrane. The concentration of DEHP in the filtered solution was detected using gas chromatography-mass spectrometry (GC-MS; GC-2010, Shimadzu, Kyoto, Japan) under the following conditions: stainless steel packed column: 1 M × 3 mm I.D, fixed liquid SE-30 10% ShimaLiteW (AW-DMCS) 60–80 mesh; programmed temperature: 250 °C (0 min), increased to 290 °C at a rate of 20 °C min^−1^ (7 min); detector temperature: 270 °C; gasification temperature: 260 °C; N_2_ flow rate: 32 mL min^−1^; H_2_ flow rate: 32 mL min^−1^; air flow rate: 300 mL min^−1^; and injection volume: 3 μL.

### 2.3. Animals and Treatment

Ninety 3-week-old specific pathogen-free C57BL/6N male mice weighing 12–15 g were purchased from Beijing Vital River Laboratory Animal Technology Co., Ltd. (Beijing, China). They were fed a free diet and maintained under a temperature of 22 ± 2 °C, humidity of 55 ± 5%, and 12/12 h light/dark cycle. After a week of acclimatization, 25 mice were randomly divided into five groups (n = 5 each): 0.1% methanol control (SC), DEHP group, 50 nm PS (PS50), and DEHP combined exposure group (PS50+DEHP), 500 nm PS (PS500) and DEHP combined exposure group (PS500+DEHP), and 5 μm PS (PS5000) and DEHP combined exposure group (PS5000+DEHP). In this experiment, each compound solution was mixed on a shaker for 24 h to combine the PS and DEHP solutions completely before administration by gavage. 20 mice were randomly divided into four groups (n = 5): SC, PS50 group, PS500 group, and PS5000 group with fluorescent PS by gavage. 45 mice were randomly divided into nine groups (n = 5 each): pure water control group (C), SC, DEHP, PS50, PS500, PS5000, PS50+DEHP, PS500+DEHP, PS5000+DEHP. The dilution medium for PS and/or DEHP was 0.1% methanol in pure water. The PS dose was determined based on the estimation described by Senathirajah et al. [48], that is, people may ingest 0.1–5 g of MPs weekly. The DEHP dose was determined considering the Federal Security Administration (FSA) report that the tolerable daily intake of DEHP in the general population is 0.05 mg kg^−1^∙bw day^−1^ [49]. Based on the dose conversions for humans and mice, the PS and DEHP doses in the mice were determined to be 50 and 0.5 mg kg^−1^∙bw day^−1^, respectively, and the administration volume was 0.1 mL 10 g^−1^ bw. The mice were weighed weekly and intragastrically administered with their respective treatments according to body weight, once a day (9:00–10:00 in the morning), for 90 days.

After exposure, blood was collected from the orbit of the mice, and the supernatant was taken after centrifugation at 3000× *g* for 15 min. The brain, liver, kidney, testis, and intestine were collected and preserved in liquid nitrogen.

### 2.4. Distribution of PS in Mice

#### 2.4.1. Distribution of PS in Tissues In Vivo

The frozen tissues (brain, liver, kidney, testis, and intestine) of the control and fluorescent PS groups were embedded in OCT, sliced into 10 μm thick sections by frozen microtome (RM2245, Leica, Nussloch, Germany), and then photographed under a laser confocal microscope (TCS SP5, Leica, Nussloch, Germany).

#### 2.4.2. Quantitative Detection of PS Distribution In Vivo

The content of fluorescent PS in the mice was detected using fluorescence colorimetry. After a standard curve was generated using fluorescence-labeled PS, 0.1 g of frozen tissue (brain, liver, kidneys, testis, and intestine) and 100 μL of serum in the SC and fluorescent PS groups were homogenized using a high-flux tissue grinder (TJG-25, Tianjin Dongfangtianjing, Tianjin, China), and the content of fluorescent PS in the tissue and serum was detected using a fluorescence enzyme-labeling instrument (Molecular Devices M5E, Silicon Valley, CA, USA).

### 2.5. Quantitative Detection of DEHP and MEHP Distribution in Mice

The brain, liver, kidneys, testis, and intestine were washed with deionized water and ether. Briefly, 0.02 g of each sample and 200 μL of serum were weighed and placed in a 2.5 mL wide-mouth conical bottle. Each sample was added with 30 mL of methanol and then placed in a KQ-500B ultrasonic cleaner. After ultrasonic extraction for 20 min, the solvent was filtered and dried, and the residue was transferred to a 10 mL volumetric flask with methanol. The injection volume was 3 μL, and GC-MS was performed under the same detection conditions described in Section 2.2.

### 2.6. Enzyme-Linked Immunosorbent Assay (ELISA)

The levels of interleukin (IL)-6, IL-1β, tumor necrosis factor α (TNF-α), and C-reactive protein (CRP) in the serum were measured using ELISA kits (Suzhou, China) according to the manufacturer’s instructions, with the absorbances detected at 450 nm using an enzyme label analyzer (Molecular Devices M5E, Silicon Valley, CA, USA).

### 2.7. In Vitro Cell Culture

In the in vitro cell model, PS50 combined with MEHP, an active metabolite of DEHP, was used to simulate compound exposure in vivo. Two types of nerve cells, mouse hippocampal neurons HT22 cells and mouse brain microglia BV2 cells (Pricella, Wuhan, China), were cultured in Dulbecco’s Modified Eagle’s Medium (DMEM; Gibco, CA, USA) supplemented with 10% fetal bovine serum (FBS; Invigentech, CA, USA) and 1% penicillin-streptomycin (Solarbio, Beijing, China) and then incubated at 37 °C in a CO_2_ atmosphere.

### 2.8. Detection of Cell Viability Using CCK8 Assay

A 100 μL aliquot of the HT22 or BV2 cell suspension was placed in a 96-well plate at a density of 5 × 10^3^ cells per well and then incubated for 24 h at 4 °C. The original 50 mg mL^−1^ PS solution in deionized water and 50 mg mL^−1^ MEHP solution in methanol were prepared. The desired concentration was prepared with 2% FBS complete medium. The concentration of the 50 nm PS solution was set to 0, 25, 50, 100, 200, 400, or 800 mg L^−1^, and the concentration of the MEHP solution was set to 0, 50, 100, 200, 400, 600, or 800 μM. The 800 µM MEHP solution contained 0.02% methanol; thus, we set 0.02% methanol as the solvent control (SC) group in the cell viability experiment. For the combined exposure to PS and MEHP, the mixed solution was placed on a shaker at 4 °C for 24 h to ensure its complete adsorption. After adding PS50, MEHP, and PS50+MEHP reagent for 24 h, each well was added with 10 μL of CCK8 solution (Invigentech, CA, USA) and then incubated for 0.5–4 h. The absorbance was determined using an enzyme-labeling instrument (Molecular Devices M5E, Silicon Valley, CA, USA) at a wavelength of 450 nm. Based on CCK8 assay results, we selected 200 mg L^−1^ PS50 and 100 μM MEHP for follow-up experiments. No difference in toxicity was found between the control and solvent control (SC) groups; thus, only the SC group was set as the control in the follow-up experiments. The two cell lines were divided into four groups: 0.0025% methanol control (SC), PS50, MEHP, and PS50+MEHP. The dilution medium for PS and/or MEHP was methanol with the same concentration in DMEM.

### 2.9. Confocal Observation of PS Uptake by Cells

The fluorescent 200 mg L^−1^ PS50 was used for this experiment. The cell climbing piece was placed in a six-well plate and then cultured. After the intervention, 100 μL DAPI was added for staining, placed at room temperature for 10 min, and PBS was used three times. The cell climbing piece was observed and photographed under a laser confocal microscope.

### 2.10. GC-MS Detection of MEHP Uptake by Cells

The cultured cells were collected after the treatment, and GC-MS detection was performed as described in Section 2.5. 

### 2.11. Statistical Analysis

Data are expressed as mean ± standard deviation (SD). Statistical analysis was performed using SPSS 26.0, normality of data was assessed by using the Shapiro–Wilks test, and homogeneity of variance was assessed by using Levene’s test. Differences between groups were analyzed by one-way analysis of variance (ANOVA). Tukey’s test was used if the variance was uniform, and Dunnett’s T3 test was used if the variance was uneven. Statistical significance was considered at *p* < 0.05. Data were processed and plotted using GraphPad Prism 9.0.

## 3. Results

### 3.1. Characteristics of PS

**Figure 1:**
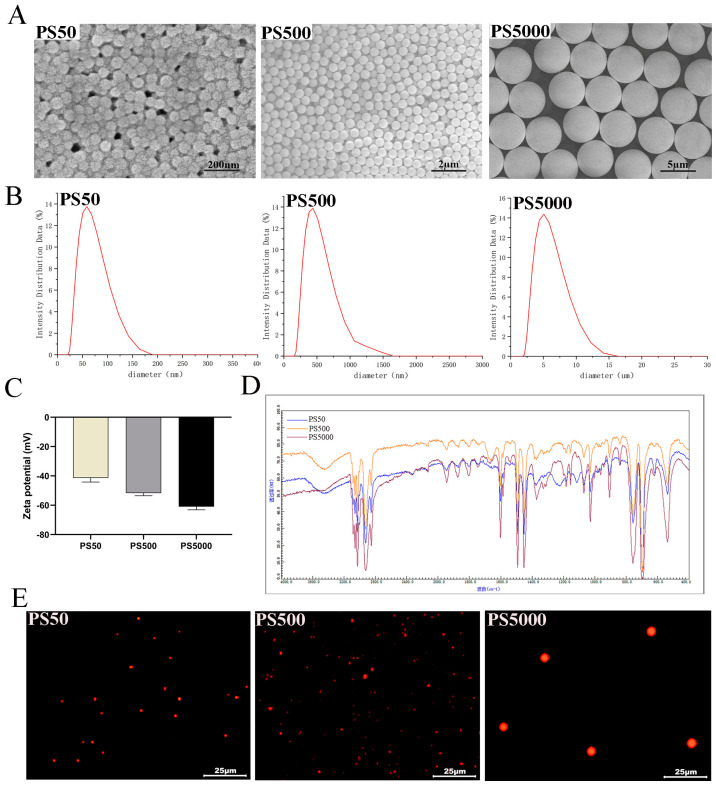
Characterization of PS: (**A**) Scanning Electron Microscope (SEM) images of PS; (**B**) hydrodynamic diameter (d_h_) of PS; (**C**) Zeta−potential analysis of PS; (**D**) Fourier transform infrared spectroscopy (FTIR) images of PS; (**E**) Fluorescence PS confocal image.

As shown in Figure 1A, PS had a regular and round shape and uniform particle size, with average particle sizes of 50 nm, 500 nm, and 5 µm. The mean d_h_ were 61.35 ± 6.02 nm for PS50, 515.15 ± 4.47 nm for PS500, 5092.50 ± 55.60 nm for PS5000 (Figure 1B). Moreover, PS has a surface negative charge in purified water with −41.39 ± 2.78 mV for PS50, −51.71 ± 1.81 mV for PS500, and −60.95 ± 2.14 mV for PS5000. Zeta potentials (>+30 mV or <−30 mV) are considered stable [50]. Therefore, both PS were considered stable in purified water (Figure 1C). Figure 1D shows the FTIR spectra of PS without any plastic plasticizer. The laser confocal micrographs in Figure 1E confirmed that the fluorescence-labeled PS could emit stable red fluorescence. 

### 3.2. In Vitro Adsorption of DEHP by PS

The DEHP standard curve is shown in Figure S1. DEHP was not detected in each group, and its concentration was lower than the lower detection limit (1 mg L^−1^). Thus, the adsorption amount of DEHP was more than 98%. In other words, DEHP was almost completely adsorbed by PS under the combined exposure concentrations of PS and DEHP.

### 3.3. Distribution of PS in Mice 

**Figure 2:**
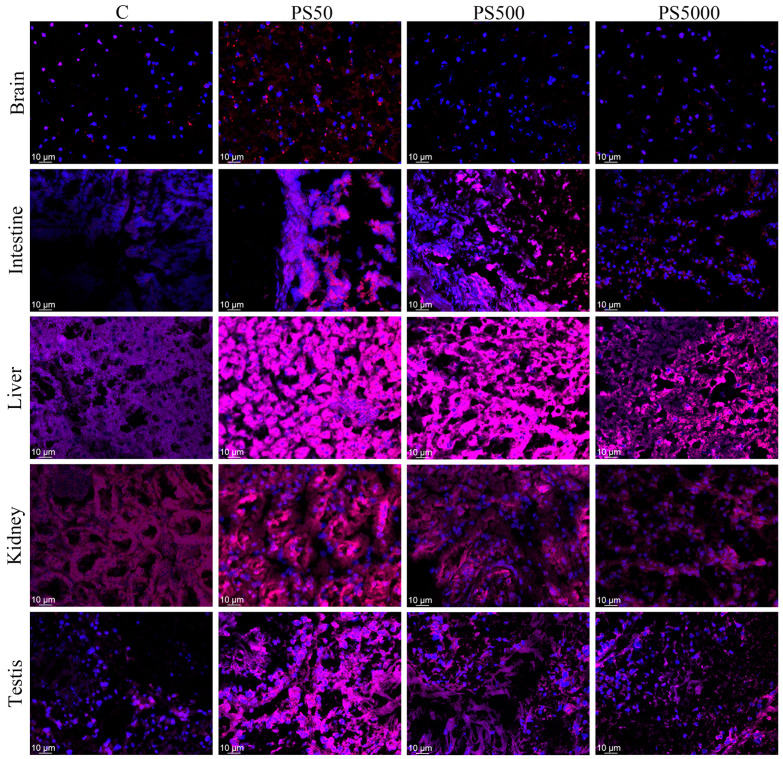
PS was distributed in various tissues of mice (n = 5). Fluorescence distribution of PS in the brains, intestines, livers, kidneys, and testes. Blue represents DAPI, and red represents fluorescence.

Confocal micrographs of the distribution of fluorescent PS in the brains, intestines, livers, kidneys, and testes of the mice are shown in Figure 2 and Figure S2. Fluorescent PS was detected in the brains, intestines, livers, kidneys, and testes of the mice. This suggested that PS could enter these tissues. 

**Figure 3:**
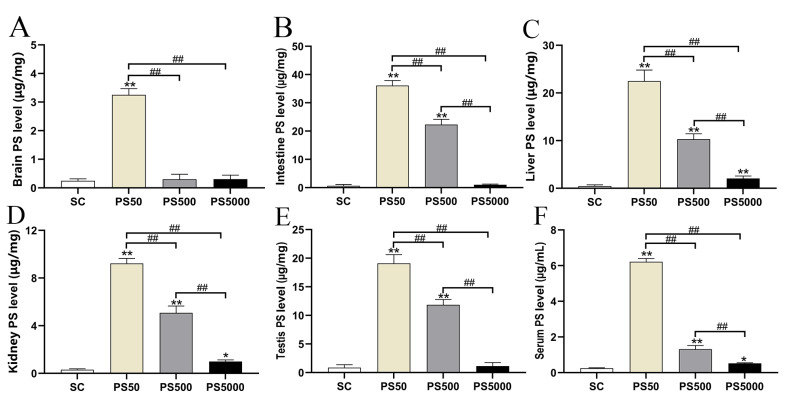
PS contents in tissues and blood of mice (n = 5). PS contents in the brain (**A**), intestine (**B**), liver (**C**), kidney (**D**), testis (**E**), and serum (**F**). * *p* < 0.05 and ** *p* < 0.01 vs. the solvent control (C) group. ## indicate *p* < 0.01.

Quantitative analysis of PS distribution in vivo showed that (Figure 3A–F) the PS contents in the brains of the mice in the PS50 group were significantly higher than those in the brains of the mice in the SC group (*p* < 0.01). In addition, the PS contents in the livers, kidneys, and serum significantly increased in all experimental groups (*p* < 0.05; *p* < 0.01), and the PS contents in the testes and intestines significantly increased in the PS50/500 group (*p* < 0.01). Meanwhile, the PS contents in the brains of the mice in the PS50 group were significantly higher than those in the brains of the mice in the PS500/5000 group (*p* < 0.01). The PS contents in the livers, kidneys, intestines, testes, and serum significantly decreased with increasing particle size (*p* < 0.01). With the PS content in the SC group as the background value, the increment of PS content in each treatment group is shown in Table 1. Results showed that the increment order of PS50 in each tissue was intestine > liver > testis > kidney > brain, and that of PS500 in each tissue was intestine > testis > liver > kidney > brain. PS5000 in each tissue was liver > kidney > intestine > testis > brain.

### 3.4. Distribution of DEHP and MEHP in Mice

**Figure 4:**
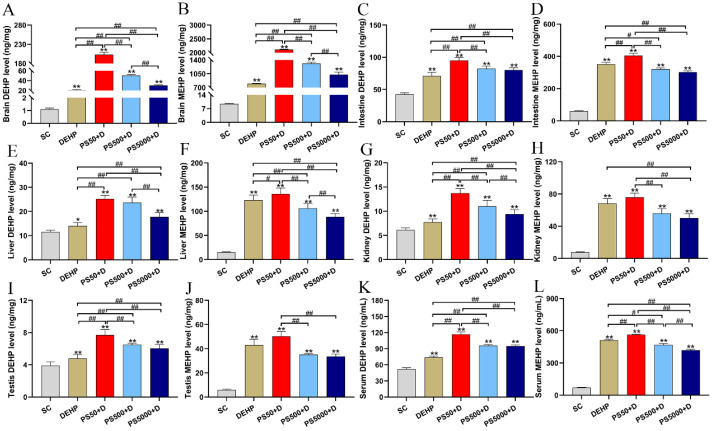
DEHP and MEHP contents in tissues and blood of mice (n = 5). DEHP and MEHP contents in the brain (**A**,**B**), intestine (**C**,**D**), liver (**E**,**F**), kidney (**G**,**H**), testis (**I**,**J**), and serum (**K**,**L**). PS50/500/5000+D represents PS50/500/5000+DEHP. * *p* < 0.05 and ** *p* < 0.01 vs. the solvent control (SC) group. # and ## indicate *p* < 0.05 and *p* < 0.01, respectively.

We detected the accumulation of DEHP and MEHP in the brains, intestines, livers, kidneys, testes, and serum (Figure 4A–L). Compared with the SC group, the DEHP group and all PS and DEHP combined exposure groups showed significantly higher contents of DEHP and MEHP (*p* < 0.05; *p* < 0.01). Compared with the DEHP group, all PS+DEHP groups had significantly higher DEHP content (*p* < 0.01). The MEHP contents in the brain, intestines, liver, and serum of the mice in the PS50+DEHP group were significantly higher than those of the mice in the DEHP group (*p* < 0.05; *p* < 0.01). The MEHP content in the brain was significantly higher in the PS500+DEHP and PS5000+DEHP groups than in the DEHP group (*p* < 0.01), whereas the MEHP contents in the intestines, liver, and serum were significantly lower in the PS500+DEHP and PS5000+DEHP groups than in the DEHP group (*p* < 0.05; *p* < 0.01). The MEHP content in the kidneys was significantly lower in the PS5000+DEHP group than in the DEHP group (*p* < 0.01). In terms of particle size, the contents of brain DEHP/MEHP, kidneys DEHP, and liver and serum MEHP significantly decreased with increasing particle size (*p* < 0.01). The contents of kidney MEHP, intestine DEHP/MEHP, serum DEHP, and testis DEHP/MEHP were significantly higher in the PS50 group than in the PS500/5000 group (*p* < 0.01). The content of liver DEHP was significantly higher in the PS50/500 group than in the PS5000 group (*p* < 0.01).

The increments of DEHP and MEHP contents in each experimental group are shown in Table 2 and Table 3. The increment of DEHP content in the DEHP and PS5000+DEHP groups was intestine > brain > liver > kidney > testis, whereas that in the PS50+DEHP and PS500+DEHP groups was brain > intestine > liver > kidney > testis. The increase in MEHP content in the PS and DEHP groups was brain > intestine > liver > kidney > testis. In addition, the increment of MEHP content in each organization was significantly higher than that of DEHP content.

### 3.5. Combined Exposure to PS and DEHP Induces Systemic Inflammation

**Figure 5:**
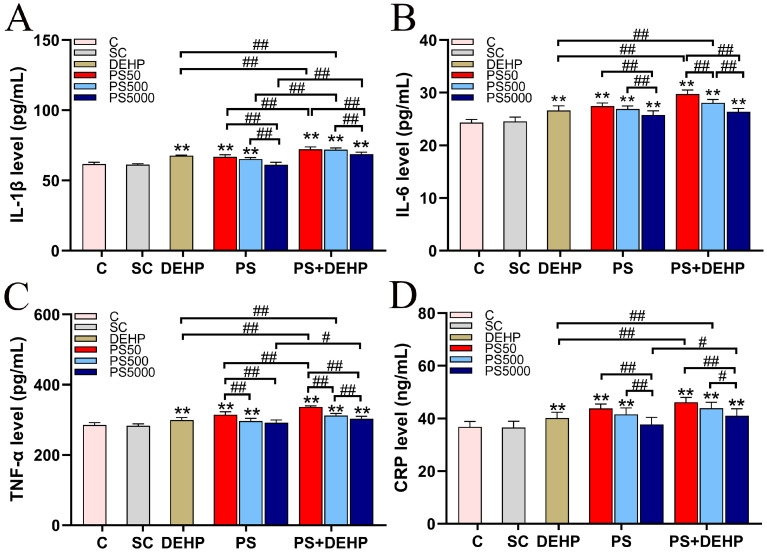
PS, DEHP, and a combination of both increased inflammation in the serum (n = 5). Levels of IL-1β, IL-6, TNF-α and CRP (**A**–**D**). PS50/500/5000+D represents PS50/500/5000+DEHP. ** *p* < 0.01 vs. the solvent control (SC) group. # and ## indicate *p* < 0.05 and *p* < 0.01, respectively.

We detected the levels of the serum inflammatory factors IL-1β, IL-6, TNF-α, and CRP (Figure 5A–D). There were no significant differences in these inflammatory factors between the C and SC groups, indicating that 0.1% methanol had no significant effect on these inflammatory factors. Compared with those in the SC group, the levels of these four inflammatory factors in the treatment group increased significantly (*p* < 0.01), except PS5000 group. The levels of these four inflammatory factors in the PS50/500+DEHP groups were also significantly higher than those in the DEHP alone group (*p* < 0.01), the levels of IL-1β in the PS+DEHP group, TNF-α in the PS50/5000+DEHP groups and CRP in the PS5000+DEHP group were also significantly higher than those in the PS alone group (*p* < 0.05; *p* < 0.01). In addition, the PS50+DEHP group had the highest levels of these four inflammatory factors, indicating that 50 nm PS and DEHP had a stronger induction effect on inflammation.

### 3.6. Effects of PS50 and MEHP Exposure Alone and in Combination on the Viability of HT22 and BV2 Cells

**Figure 6:**
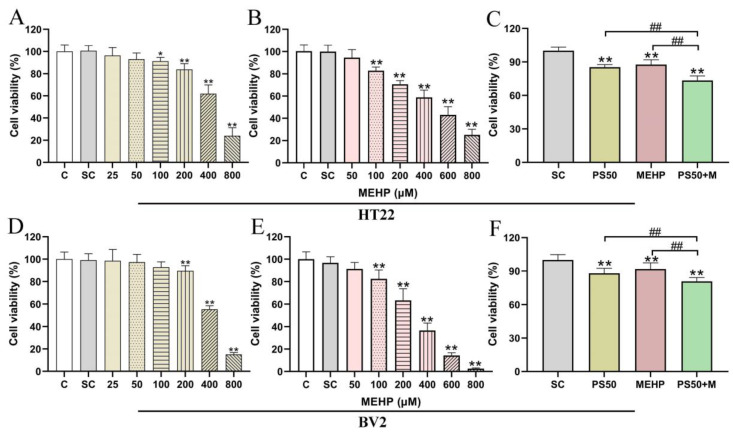
Effects of PS, MEHP, and their combined exposure on the viabilities of HT22 and BV2 cells (n = 5). (**A**–**C**) Survival rate of HT22 cells exposed to different concentrations of PS50 and MEHP alone or in combination; (**D**–**F**) Survival rate of BV2 cells exposed to different concentrations of PS50 and MEHP alone or in combination. * *p* < 0.05 and ** *p* < 0.01 vs. solvent control (SC) group. PS50+M represents PS50+MEHP ## indicate *p* < 0.01.

We measured the viabilities of HT22 (Figure 6A–C) and BV2 (Figure 6D–F) cells exposed to different concentrations of PS50 and MEHP for 24 h. The survival rates of the HT22 and BV2 cells gradually decreased with increasing exposure doses of 50 nm PS and MEHP alone. Significant differences were found under the exposure to 200, 400, and 800 mg L^−1^ PS50 and 100, 200, 400, 600, and 800 μM MEHP (*p* < 0.01). Therefore, the concentrations yielding a cell survival rate of approximately 80% were selected for the follow-up experiments with 200 mg L^−1^ PS50 and 100 μm MEHP. Meanwhile, the survival rates of the HT22 and BV2 cells were significantly lower in the PS50+MEHP group than in the SC, PS50, and MEHP groups (*p* < 0.01).

### 3.7. Accumulation of PS and MEHP in HT22 and BV2 Cells

**Figure 7:**
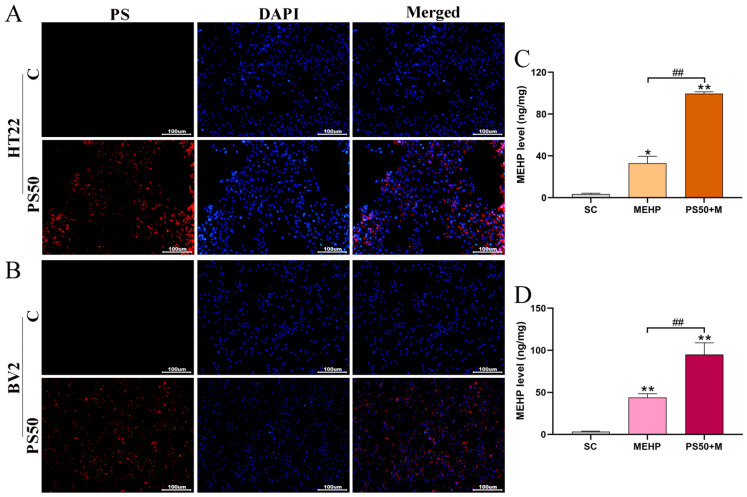
Accumulation of PS and MEHP in HT22 and BV2 cells (n = 5). (**A**,**B**) Fluorescence PS50 accumulation in HT22 and BV2 cells. Red fluorescence represents PS50, and blue fluorescence represents nuclear staining. (**C**,**D**) MEHP content in HT22 and BV2 cells. * *p* < 0.05 and ** *p* < 0.01 vs. the solvent control (SC) group. ## indicate *p* < 0.01.

To detect whether 50 nm PS can enter HT22 and BV2 cells, we added fluorescent 50 nm PS to the culture medium. We observed the PS uptake by the cells under a confocal microscope (Figure 7A,B). The fluorescence in the HT22 and BV2 cells enhanced after exposure to fluorescent 50 nm PS and 50 nm PS could enter the nucleus. To evaluate and quantify the uptake of MEHP by the HT22 and BV2 cells, we assayed the MEHP content in the HT22 and BV2 cells (Figure 7C,D). Results showed that the MEHP contents in the HT22 and BV2 cells were significantly higher in the MEHP and PS50+MEHP groups than in the SC group (*p* < 0.05; *p* < 0.01), and the combined exposure group had significantly higher MEHP contents than the MEHP group (*p* < 0.01).

## 4. Discussion

Due to the high surface hydrophobicity of MNPs, especially NPs, and their high surface area, MNPs have become potential carriers for various pollutants [51]. DEHP is a plasticizer widely used in the production of plastic products. The combination of MNPs and DEHP is not covalent and can be released during the aging and degradation of plastics [52]. Therefore, we selected the exposure concentrations of PS and DEHP based on their environmental concentrations and carried out the adsorption experiment. As a result, more than 98% of DEHP was adsorbed by PS, which provided a theoretical basis for further experiments on the enrichment of PS-carried DEHP in various organs.

Xu et al. found an accumulation of 100 nm PS in mice kidneys, intestines, testes, and brains [53]. Ding et al. found that PS accumulates in mice’s intestines, kidneys, and liver when orally administered with 60 nm PS [54]. We also examined the distribution of fluorescent PS in mice to assess the risk of human PS enrichment in vivo. This experiment found fluorescent PS in mice brains, intestines, livers, kidneys, and testes under a confocal microscope. We also quantitatively detected the content of fluorescent PS in various tissues. PS50, PS500, and PS5000 were enriched in the liver, kidneys, and serum. 

PS50 was enriched in the brain, whereas PS50 and PS500 PS were enriched in the intestines and testis. In this study, the accumulation of 500 nm and 5 μm PS in the brain and 5 μm PS in the intestines and testis was not observed. These results may be ascribed to the fact that the particle size is too large to penetrate the blood–brain and blood–testis barriers, considering that particle size is a crucial factor in determining the biological distribution of particles in various organs [55]. Liang et al. found that both 50 and 500 nm PS were accumulated in the brain, intestine, liver, kidney, testis, and blood after mice were orally administrated with 50 nm and 500 nm PS for 24 h [56]. Based on our results, we speculated that 50 nm PS could cross the blood–brain barrier (BBB) upon oral exposure for 90 days; 50 and 500 nm PS were able to cross the blood–testis barrier. Deshmukh et al. found that aggregated nanogel particles larger than 3 μm and less than 10 μm are temporarily trapped in the lungs and then transferred to the liver [57]. Therefore, 5 μm PS may not accumulate in the intestines. Except for the brain, the content of the other tissues significantly decreased with increasing particle size.

The accumulation of PS in mice may be related to particle size, and the biological interaction of nanoparticles may affect membrane permeability [58], allowing them to easily enter the bloodstream and transfer to other tissues through blood circulation [16,59,60]. This phenomenon increases the biological distribution and, consequently, the toxicity of nanoparticles. Comparison of the increment of PS with different particle sizes in different tissues showed that PS500 and PS50 accumulated most in the intestines, whereas 5 µm PS accumulated in the gastrointestinal tract and then transferred to other tissues [61]. In the present study, PS5000 accumulated most in the liver, consistent with Deshmukh et al.’s results [57]. Thus, it may be mainly metabolized in the liver, but its specific mechanism warrants further investigation.

PS can adsorb DEHP and carry it into the body. After DEHP enters the body, one part will be absorbed directly, and pancreatic and intestinal enzymes will degrade the other. MEHP is the primary metabolite of DEHP, mainly in the intestines, but it can be degraded in other tissues, such as the liver, kidneys, and testis. Part of DEHP will be excreted within 24 h, whereas MEHP can accumulate in human tissue for up to 6 months [62]. Therefore, we detected the accumulation of DEHP and MEHP in each tissue and found that exposure to DEHP and PS+DEHP can cause a significant accumulation of DEHP and MEHP in each tissue. However, this accumulation decreased with increasing particle size. This result can be attributed to the fact that more small PS particles can enter the tissue and thus carry more DEHP. Deng et al. observed that MPs could transport and release the PAEs to the intestinal tract of mice [61], suggesting that DEHP adsorbed by PS might be dissociated in vivo. DEHP needs to be resolved from PS before being biodegraded. Thus, the DEHP detected in tissue is DEHP adsorbed on PS and that which has not been degraded and utilized. MEHP may be present in tissues because DEHP that has not been decomposed and utilized by the organism is metabolized into MEHP by the organism. The DEHP content in the PS+DEHP group was significantly higher than that in the DEHP group, which was consistent with the finding of Deng et al. that PS can adsorb PAEs, carry PAEs to the intestines, and cause intestinal accumulation [63]. Therefore, PS can adsorb DEHP; carry DEHP to the blood, brain, intestines, liver, kidneys, and testis; and cause DEHP accumulation in various tissues.

The highest increase in brain DEHP content was found in the PS50/500+DEHP group, whereas the highest increase in intestine DEHP was found in the DEHP and PS5000+DEHP groups. This result may be ascribed to the blood–brain barrier in the brain, which prevented DEHP carried by PS5000 from entering the brain. Furthermore, the MEHP content in all tissues was higher in the PS50+DEHP group than in the DEHP group but lower in the PS500/5000+DEHP group than in the DEHP group. This result may be attributed to tissue hydrolysis and metabolism. In addition, the highest increment of MEHP content was achieved in the brain, suggesting that the brain is an important target organ. Moreover, the content of MEHP in all tissues was significantly higher than that of DEHP. Considering that MEHP has a higher biological toxicity than DEHP [39,40], we speculate that PS and MEHP play a significant role in the toxicity caused by the joint exposure of PS and DEHP.

When the body receives external stimuli, such as stimulation of compounds, it will secrete cytokines to deal with it and promote the production of inflammatory factors. IL-1β, IL-6, TNF-α, and CRP are important pro-inflammatory factors. Here, PS exposure alone and in combination with DEHP resulted in significantly higher serum IL-6, IL-1β, TNF-α, and CRP levels than did the controls, consistent with previous studies reporting that exposure to 1 μm PS and DEHP alone increased inflammation factors in the serum of rats or mice [64,65]. In addition, combined exposure to PS and DEHP, especially that with 50 nm PS, induces a more severe inflammatory response. Results of in vivo distribution showed that PS50 could significantly accumulate in the brain. Thus, a cell simulation experiment of combined exposure to PS-loaded DEHP was conducted in vitro. We chose PS50 and two important nerve cells: neurons and microglia. These two kinds of nerve cells were used to simulate the uptake of PS and DEHP after combined exposure in vivo. MEHP is used for in vitro exposure instead of DEHP because it exerts a toxic effect in vivo, mainly its active metabolite MEHP [30,31]. The cells cultured in vitro cannot activate DEHP metabolism. Thus, we used PS and MEHP to expose cells in vitro and simulate the biotoxicity in vivo.

We determined the exposure doses of PS and MEHP to the cells by conducting a cell activity test. Results showed that the activities of the HT22 and BV2 cells decreased with increasing PS and MEHP exposure concentrations. Thus, we used 200 mg L^−1^ PS50 and 100 µM MEHP for combined exposure. Results showed that exposure to PS50 and MEHP alone and in combination could affect the activities of the mouse HT22 and BV2 cells. Compared with their single exposure, the combined exposure of PS and MEHP resulted in significantly lower survival rates of HT22 and BV2 cells. In addition, the cytotoxicity of PS combined with MEHP was stronger than that of PS or MEHP alone.

Confocalization showed PS50 entered the HT22 and BV2 cells and significantly increased MEHP content in the cells. Thus, PS50 adsorbed and carried MEHP into the HT22 and BV2 cells, which increased MEHP content and exacerbated cytotoxicity.

One limitation of the present study is that the adsorption–dissociation state of PS-carried DEHP into the body could not be traced. Thus, the metabolic pattern of the compound exposure in vivo needs more in-depth analysis. This paper may serve as a reference for the health risk assessment of combined exposure to MNPs and environmental pollutants.

## 5. Conclusions

All three particle sizes of PS were able to enter the blood system of the mice. DEHP carried to the body was metabolized to MEHP, which was much higher than DEHP and more likely to be enriched in the brain. The accumulation of MEHP in the brain significantly increased after combined exposure to PS and DEHP, suggesting for the first time that the brain is an important target organ for the toxic effects of combined exposure to PS and DEHP. The levels of inflammatory factors in the serum significantly increased after combined exposure to PS and DEHP, suggesting that combined exposure to PS and DEHP causes systemic inflammation. The PS content alone, DEHP/MEHP content, and inflammatory factors levels increased with decreasing particle size after PS+DEHP exposure. In addition, PS50 carried MEHP into neuronal cells. This study provides clues to evaluate the neurotoxicity caused by combined PS and DEHP exposure to the organism and its related mechanisms. It may serve as a reference for the health risk assessment of combined exposure to MNPs and environmental pollutants.

## Figures and Tables

**Table 1:** Increment in each tissue after exposure to PS with different particle sizes (n = 5).

Group	Brain (μg mg^−1^)	Intestine (μg mg^−1^)	Liver (μg mg^−1^)	Kidney (μg mg^−1^)	Testis (μg mg^−1^)
PS50	3.0053	35.4827	22.026	8.9173	18.2173
PS500	0.0533	21.6607	9.814	4.7713	10.9733
PS5000	0.0613	0.3767	1.588	0.6833	0.2633

Values in the table refer to the difference between the PS content in each group and the solvent control group.

**Table 2:** Increment of DEHP in various tissues of mice (n = 5).

Group	Brain (ng mg^−1^)	Intestine (ng mg^−1^)	Liver (ng mg^−1^)	Kidney (ng mg^−1^)	Testis (ng mg^−1^)
DEHP	19.39	28.33	2.41	1.62	0.87
PS50+D	201.53	52.55	13.66	7.62	3.81
PS500+D	49.28	39.62	12.08	4.93	2.59
PS5000+D	29.24	37.58	6.21	3.26	2.14

Values in the table refer to the difference between the DEHP content in each group and the solvent control group.

**Table 3:** Increment of MEHP in various tissues of mice (n = 5).

Group	Brain (ng mg^−1^)	Intestine (ng mg^−1^)	Liver (ng mg^−1^)	Kidney (ng mg^−1^)	Testis (ng mg^−1^)
DEHP	789.04	291.81	108.04	60.50	37.26
PS50+D	2099.64	345.60	120.98	68.06	44.40
PS500+D	1303.59	260.28	91.21	48.08	29.17
PS5000+D	1021.50	241.99	73.47	42.14	27.71

Values in the table refer to the difference between the MEHP content in each group and the solvent control group.

## Supplementary Materials

The following supporting information can be downloaded at: https://www.mdpi.com/article/10.3390/toxics11050441/s1, Figure S1: DEHP standard curve. Figure S2: PS was distributed in various tissues of mice.
