# Supplementary figures and images for: Distribution of Micro-Nano PS, DEHP, and/or MEHP in Mice and Nerve Cell Models In Vitro after Exposure to Micro-Nano PS and DEHP
Source: Toxics. 2023 May 7;11(5):441. doi: 10.3390/toxics11050441 (PMC10220640; doi:10.3390/toxics11050441)

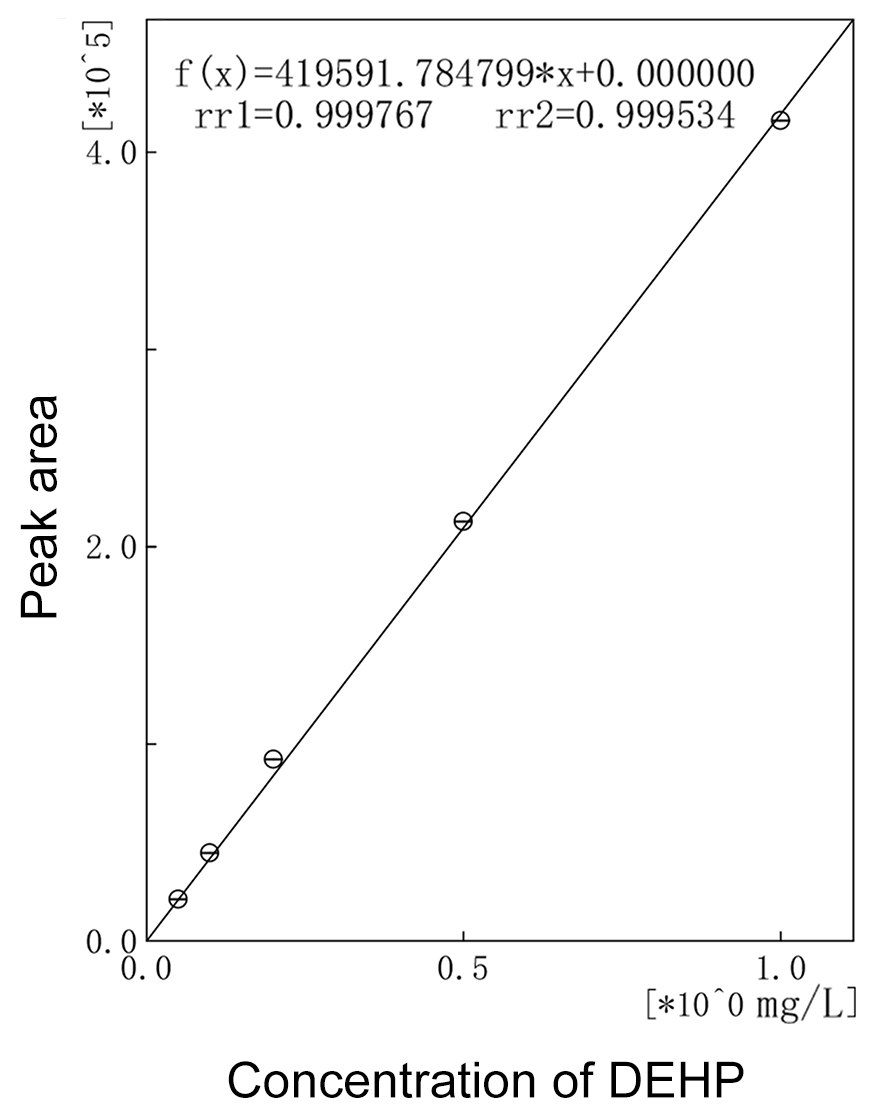

Supplement: Supplementary file 1 [file toxics-11-00441-s001.zip › Figure S1.tif]

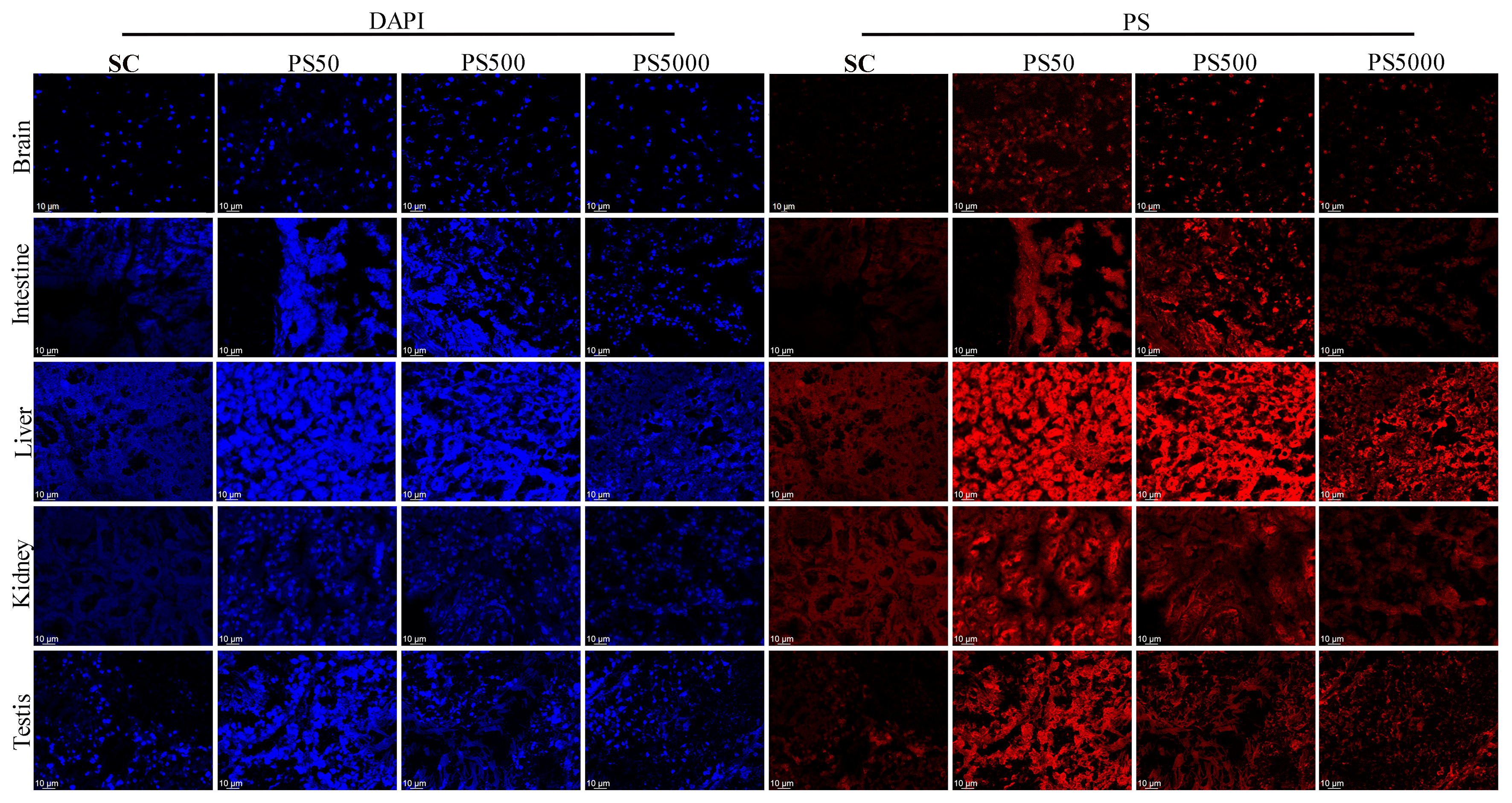

Supplement: Supplementary file 1 [file toxics-11-00441-s001.zip › Figure S2.tif]
